# A Role for Immune Responses against Non-CS Components in the Cross-Species Protection Induced by Immunization with Irradiated Malaria Sporozoites

**DOI:** 10.1371/journal.pone.0007717

**Published:** 2009-11-05

**Authors:** Marjorie Mauduit, Anne Charlotte Grüner, Rita Tewari, Nadya Depinay, Michèle Kayibanda, Jean-Marc Chavatte, Jean-François Franetich, Andrea Crisanti, Dominique Mazier, Georges Snounou, Laurent Rénia

**Affiliations:** 1 Singapore Immunology Network (SIgN), Agency for Science, Technology and Research (A*STAR), Biopolis, Singapore; 2 Department of Immunology, Institut Cochin, Université Paris Descartes, CNRS (UMR 8104), Paris, France; 3 INSERM, U567, Paris, France; 4 Division of Cell and Molecular Biology, Faculty of Natural Sciences, Imperial College, London, United Kingdom; 5 Institute of Genetics, School of Biology, University of Nottingham, Nottingham, United Kingdom; 6 Parasitologie Comparée et Modèles Expérimentaux USM0307, CNRS IFR101, Muséum National d'Histoire Naturelle, Paris, France; 7 INSERM U945, Paris, France; 8 Université Pierre et Marie Curie-Paris6, UMR S945, Paris, France; 9 Assistance Publique Hopitaux de Paris (AP HP), Groupe Hospitalier Pitié-Salpêtrière, Service parasito-Mycologie, Paris, France; Federal University of São Paulo, Brazil

## Abstract

Immunization with irradiated *Plasmodium* sporozoites induces sterile immunity in rodents, monkeys and humans. The major surface component of the sporozoite the circumsporozoite protein (CS) long considered as the antigen predominantly responsible for this immunity, thus remains the leading candidate antigen for vaccines targeting the parasite's pre-erythrocytic (PE) stages. However, this role for CS was questioned when we recently showed that immunization with irradiated sporozoites (IrrSpz) of a *P. berghei* line whose endogenous CS was replaced by that of *P. falciparum* still conferred sterile protection against challenge with wild type *P. berghei* sporozoites. In order to investigate the involvement of CS in the cross-species protection recently observed between the two rodent parasites *P. berghei* and *P. yoelii*, we adopted our gene replacement approach for the *P. yoelii* CS and exploited the ability to conduct reciprocal challenges. Overall, we found that immunization led to sterile immunity irrespective of the origin of the CS in the immunizing or challenge sporozoites. However, for some combinations, immune responses to CS contributed to the acquisition of protective immunity and were dependent on the immunizing IrrSpz dose. Nonetheless, when data from all the cross-species immunization/challenges were considered, the immune responses directed against non-CS parasite antigens shared by the two parasite species played a major role in the sterile protection induced by immunization with IrrSpz. This opens the perspective to develop a single vaccine formulation that could protect against multiple parasite species.

## Introduction

Sporozoites inoculated by the mosquito must invade and develop within hepatocytes in order to generate merozoites that can then initiate the pathogenic erythrocytic phase. Thus, this obligatory transient phase of the life cycle is an attractive target for interventions to inhibit parasite development fully, as this would prevent both disease and transmission. Sterile immunity against pre-erythrocytic (PE) stages is an all-or-none phenomenon, because merozoites produced by a single infected hepatocyte would lead to a patent blood infection. Immunization with large numbers of radiation-attenuated sporozoites has long been the only protocol that led to the induction of sterile immunity in rodents and humans [1], [2]. Subsequent investigations using the rodent malaria parasites, *P. berghei* and *P. yoelii*, revealed a role for both humoral and cellular immune responses targeting the sporozoite and the infected hepatocyte, respectively [3]. In the vaccinated hosts the antibody responses induced are predominantly directed against the antigenic repetitive central domain of circumsporozoite protein (CS) [4]. Additionally adoptive transfer of CS-specific CD8^+^ or CD4^+^ T cell clones, albeit in large numbers, could lead to full protection against sporozoite challenge [5]–[7]. Together these observations have led the CS to be considered as the parasite antigen responsible for the sterile protection induced by IrrSpz. This view was recently reinforced by a report that concluded that CS is a protective immunodominant antigen from experiments where mice made tolerant to CS of *P. yoelii* were less likely to develop protective immune responses when immunized with *P. yoelii* IrrSpz [8]. However, this conclusion is mitigated by the demonstration in the same study that sterile protection did actually develop when three rather than two injections of IrrSpz were used to immunize the CS-tolerant transgenic mice [8], [9]. Further indications that sterile protection can be obtained independently of immune responses to the CS were obtained when immunization with *P. berghei* IrrSpz whose endogenous CS was replaced by that of *P. falciparum* fully protected mice from challenge with wild type *P. berghei* sporozoites [10].

It had been recently suggested that anti-CS responses might be implicated in the cross-species protection that has been observed between *P. yoelii* and *P. berghei* in the context of IrrSpz immunization [11], possibly because of the extensive sequence homology between the N- and C-terminal of their CS because the repeat regions are quite distinct (Figure S1). Indeed, adoptive transfer of a CD8^+^ T cell clone specific for the *P. yoelii* CS CD8^+^ immunodominant epitope protected mice from challenge with *P. berghei* sporozoites [12]. In order to investigate the actual role of immune responses induced against the CS in cross-species sterile protection we exploited the gene replacement approach [13] to generate *P. berghei* sporozoites expressing the CS of *P. yoelii* (*P. berghei* [PyCS]) instead of the endogenous CS, for use with those of wild type *P. berghei* and *P. yoelii* in reciprocal immunization/challenge experiments. This also afforded us the opportunity to characterise the role of CS in sterile protection in the two widely used rodent models of IrrSpz immunization.

## Results

### T Cell Responses

**Figure 1:**
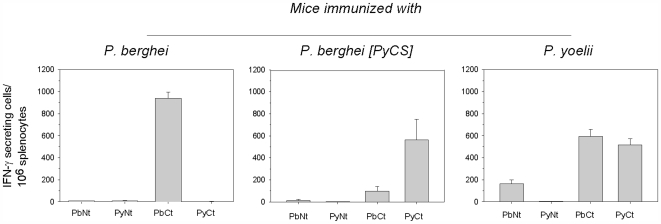
CS–specific T cells induced by immunization with irradiated sporozoites. Mice were immunized 3 times with IrrSpz from the different parasite lines. The frequency of epitope-specific CD8^+^ or CD4^+^ T cells in spleens was assessed by IFN-γ ELISPOT using long CS peptides 10 days after the last immunization. Long peptides PyNt, PbNT and PyCt, PbCt correspond to the *P. yoelii* or *P. berghei* CS N- and C-terminal region of CS, respectively. These peptides encompass potential CD4^+^ and CD8^+^ T cells epitopes. Results are expressed as the mean±SEM of epitope–specific T cells from 5 mice per group.

Groups of BALB/c mice were immunized with three doses of *P. berghei*, *P. berghei* [PyCS] or *P. yoelii* IrrSpz. Cross-reactive T cell responses induced by the immunizations were assessed by ELISPOT using long peptides corresponding to N-terminal or C-terminal regions of *P. berghei*, and *P. yoelii* CS, which contain all the potential CD4 and CD8 epitopes (Figure 1). Whereas splenic T cells from mice immunized with *P. berghei* irradiated sporozoites only recognized peptides derived from the *P. berghei* CS, those from mice immunized with *P. berghei* [PyCS] or *P. yoelii* IrrSpz also recognized the C-terminus peptides derived from *P. berghei*, in addition to the peptides derived from *P. yoelii* CS. This cross-species recognition was more substantial for mice immunized with *P. yoelii* IrrSpz, which surprisingly additionally recognized the long peptide derived from the heterologous N-terminus of the *P. berghei* CS but not of the homologous *P. yoelii* CS (Figure 1 middle and left panels). This unexpected observation, confirmed in duplicate experiments, remains as yet unexplained.

**Figure 2:**
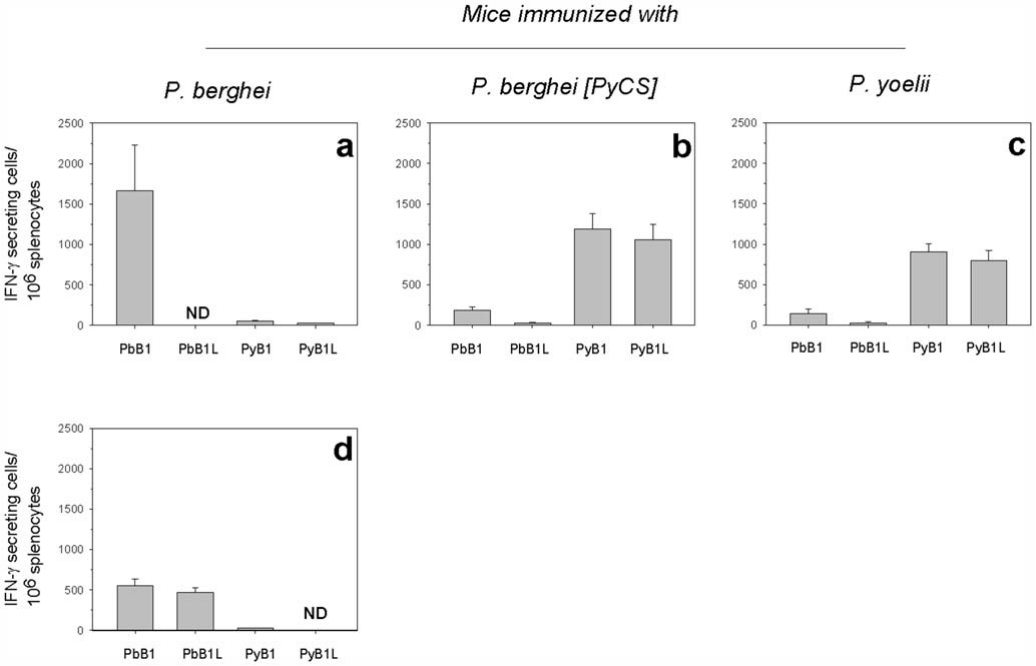
CD8+T cells specific for the CS CD8^+^ immunodominant epitopes induced by immunization with irradiated sporozoites do not cross-react. The frequency of epitope-specific CD8^+^ T cells in spleens from mice immunized 3 times with IrrSpz from *P. berghei*- (**a, d**, two separate experiments), from *P. berghei* [PyCS] (**b**) and *P. yoelii* (**c**) were assessed by IFN-γ ELISPOT using short CS peptides, 10 days after the last immunization. PbB1 and PyB1 are 9-mer peptides containing the major H2-Kd-restricted CD8 epitopes located in the same position in the C-terminal part of the CS. PbB1L and PyB1L are 19-mer peptides and correspond to extended version of PyB1 and PbB1. These peptides encompass potential CD4 and CD8 T cells epitopes. Results are expressed as the mean±SEM of epitope–specific T cells from 5 mice per group. ND, not done.

To determine if this cross-reactivity observed for the peptide derived from the C-terminus of the CS molecule were due to CD8^+^ T cells recognizing the immunodominant CD8 epitope located in this region, as previously suggested using CS-specific T cell clones [12], a set of peptides containing this immunodominant CD8 epitope (9-mer and 19-mer for *P. berghei* and 9-mer and 17-mer for *P. yoelii*) were tested by ELISPOT. This was not the case, because the cross-reactivity was found to be minimal (Figure 2a–2d).

Thus, immunization with *P. yoelii* but not *P. berghei* IrrSpz induced cross-reactive anti-CS T cells, most likely CD4^+^ T cells. The magnitude of the cross-reactivity was different depending on the context in which the *P. yoelii* CS was (i.e. whether it was expressed in a *P. berghei* or in *P. yoelii* sporozoite background).

### Antibody Responses

**Figure 3:**
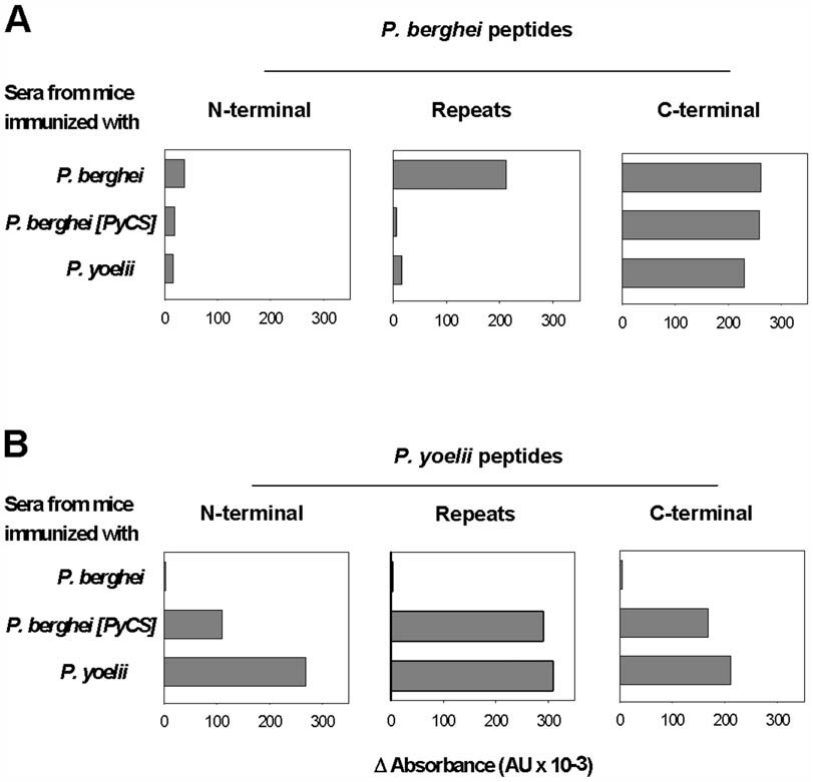
IgG antibody responses to *P. yoelii* and *P. berghei* CS domains. Pooled serum samples from groups of five mice immunized 3 times with IrrSpz from the different parasite lines were analyzed by ELISA using peptides covering domains of the *P. berghei* (**A**) or *P. yoelii* (**B**) CS. Data are expressed as differential absorbance where values from pooled normal serum were subtracted from experimental values. The data presented are representative of 2 experiments.

The levels of antibodies induced after immunization with three injections of IrrSpz from the three parasite lines were assessed by ELISA using peptides corresponding to the three domains of CS (N-terminus, repeat region and C-terminus), and by IFA using whole sporozoites. Anti-CS specific IgG and IgM induced by immunization with *P. berghei* IrrSpz were solely directed against the homologous *P. berghei* but not heterologous *P. yoelii* CS peptides (Figure 3 and Figure S4). The antibodies induced by immunization with the two other lines (*P. berghei* [PyCS] or *P. yoelii*) not only recognized their homologous CS peptides but also cross-reacted with the heterologous C-terminal peptides derived from *P. berghei* CS, with higher levels observed for IgG as compared to IgM (Figure 3A and Figure S4).

**Figure 4:**
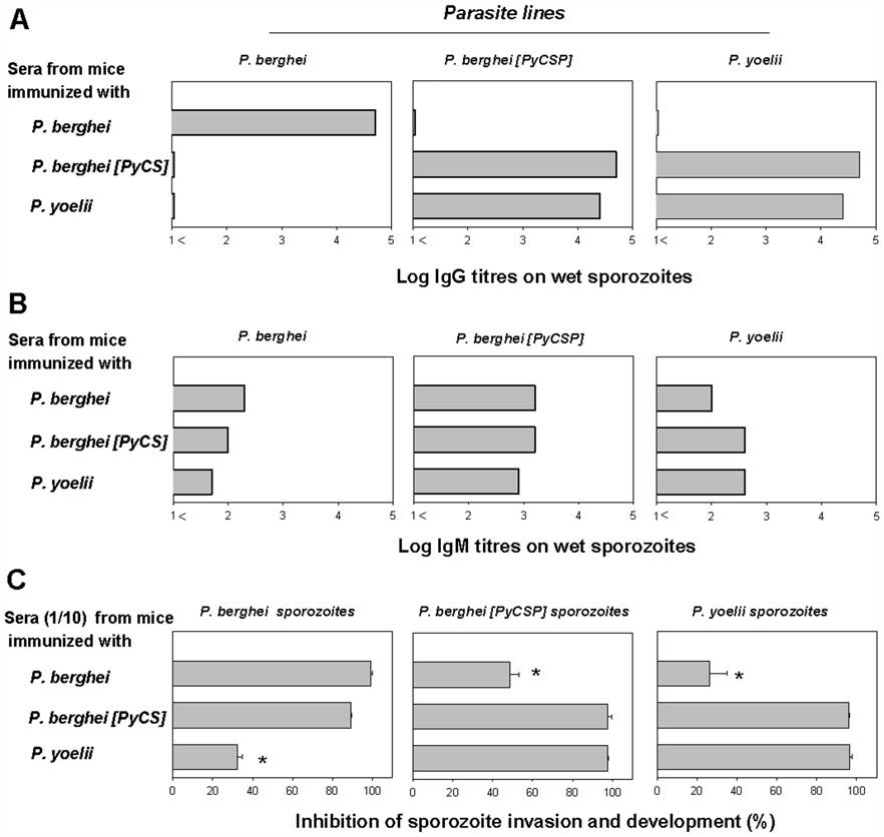
Antibody reactivity to sporozoites induced by immunization with irradiated sporozoites. (**A**) IgG responses were exclusively directed against CS. Pooled serum samples from groups of five mice immunized three times with IrrSpz from the different parasite lines were analyzed by IFA against wet sporozoites to detect surface antigens using secondary antibodies specific to the IgG. Titres are expressed as the log of the highest dilution of serum giving a positive staining. (**B**) IgM response to the CS and other sporozoite epitopes on the surface of the sporozoites. Pooled sera were tested as above using secondary antibodies specific to IgM. Titres were expressed as above. (**C**) In vitro sporozoite invasion and development inhibition assay using pooled sera (at a 1/10 dilution) from group of five mice immunized three times with IrrSpz from the different parasite lines. The data presented is representative of those obtained in duplicate experiments. Control wells tested with normal serum (1/10 dilution) contained the following numbers of parasite forms: 807±39.6 *P. berghei* schizonts, 162.7±8.1 *P. berghei* [PyCS] schizonts, and 207.3±31.3 *P. yoelii* schizonts.

Immunization with IrrSpz induced high levels of IgG against homologous but none against heterologous sporozoites. Sera from mice immunized IrrSpz had an IFA titre of ∼1/200 000 on wet homologous sporozoites (for which only surface antigens are accessible) (Figure 4A), and an IFA titre of ∼1/400 000 on dried methanol-fixed sporozoites (for which both intracellular and surface antigens are accessible) (Figure S5). By contrast, negligible IFA IgG titres (below 1/10) were obtained against the wet or the dried methanol-fixed sporozoites expressing the heterologous CS. Since sporozoites from these lines differed only for the CS, this meant that IgG were predominantly directed against the CS.

The titres of IgM responses induced against the homologous sporozoites were one to two orders of magnitude lower (corresponding to IFA titres of 1/200–1/1600) against wet sporozoites than those observed for IgG (Figure 4B). Similar results were obtained with sera obtained from animals immunized only once as opposed to three times with IrrSpz, though in this case the antibody levels were much lower (1/50–1/100).

The fact that the CS cross-reactivity of the antibodies induced by IrrSpz immunization was revealed only when peptides but not when whole sporozoites were used suggested that the cross-reactive antibodies induced by immunization with *P. yoelii* and *P. berghei* [PyCS] IrrSpz recognized epitopes that were not exposed in the CS expressed by *P. berghei* salivary gland sporozoites.

It was possible to gather some qualitative estimate of the contribution that anti-CS humoral reactivities made to the inhibition of parasite invasion and development as compared to those directed against other antigens. This was achieved in an in vitro assay where sporozoites were added in the presence of sera (used at 1/10 dilution) and the numbers of liver stage parasites that reached maturity were subsequently counted. Sera from animals immunized 3 times with IrrSpz were strongly inhibitory (>90%) to invasion and development of the homologous parasites (Figure 4C). When the heterologous combinations were similarly assayed, inhibition was also observed but it varied in intensity (Figure 4C). The sera from *P. berghei* IrrSpz-immunized mice were moderately inhibitory (30%–40%) against the heterologous *P. yoelii* sporozoites (Figure 4C, top line, right panel). Since cross-reactivities induced against *P. yoelii* sporozoites were only due to the IgM fraction and since very little cross-reactive IgM to *P. yoelii* CS peptides were detected in the sera from *P. berghei* IrrSpz-immunized mice (Figure S4), this indicated that anti-CS IgG but not IgM contributed to more than half of the inhibition measured in vitro against homologous parasites while the remaining inhibition was mediated by IgM against other non-CS antigens.

When the sera from mice immunized with *P. berghei* [PyCS] IrrSpz were tested, inhibition of *P. berghei* sporozoites was high (90%). This contrasted with a weak (30%) inhibitory activity of sera raised by *P. yoelii* IrrSpz immunization against *P. berghei* sporozoites (Figure 4C, left panel, from top to bottom). However, we could not draw meaningful conclusions as to the likely role of the anti-CS versus anti-non CS component of these inhibitory activities. This is evident when one compares the high cross-reactivity observed for IgG in ELISA against CS peptides (Figure 3) with a low cross-reactivity for the same sera when wet or air-dried methanol-fixed sporozoites were used (Figures 4 and S5). Nonetheless, other indications of the differential contribution to sterile immunity can be obtained from *in vivo* challenge studies.

### Protection Studies

**Figure 5:**
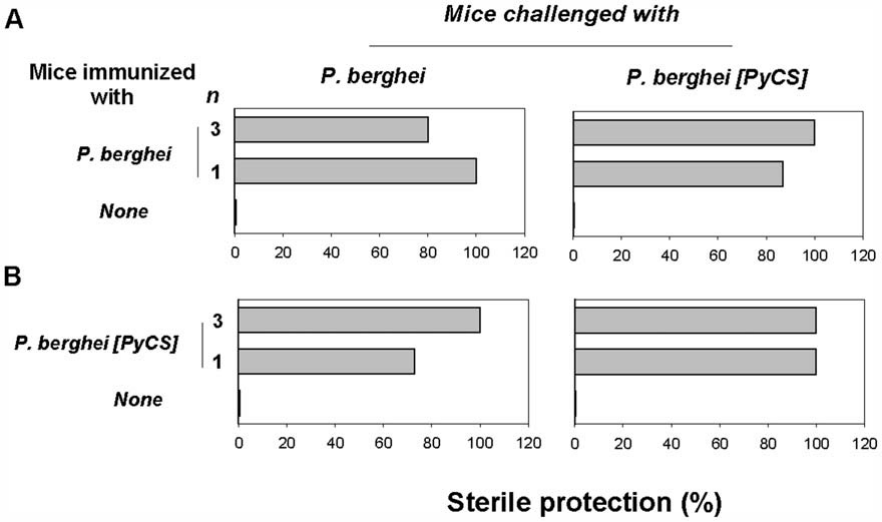
Sterile protection in mice immunized with IrrSpz and challenged with sporozoites of *P. berghei* or *P. berghei* [PyCS]. Mice were immunized with 1 or 3 injections of *P. berghei* (**A**) or *P. berghei* [PyCS] (**B**) IrrSpz and challenged with 5 000 *P. berghei* or *P. berghei* [PyCS] sporozoites at least one week after the last IrrSpz injection. All groups (5 mice per group) were monitored for blood-stage infections by examination of Giemsa-stained blood smears obtained daily from day 3 to day 10 post-challenge. All naive control mice developed a patent blood-stage infection. The data is representative of those obtained in triplicate experiments.

Sterile protection was equally observed in 80 to 100% of the BALB/c mice inoculated once or three times with *P. berghei* IrrSpz and then challenged with *P. berghei* or *P. berghei* [PyCS] sporozoites (Figure 5A). We also obtained identical results using another *P. berghei* [PyCS] clone (data not shown). Protection was not restricted to BALB/c because outbred CD1 mice immunized once or three times with *P. berghei* IrrSpz and then challenged with *P. berghei* or *P. berghei* [PyCS] were also fully protected (Figure S6). These results indicated that when *P. berghei* IrrSpz were used for immunization, they induced sterile immunity independently of the *P. berghei* CS. Next, experiments were performed to determine if this was equally true for the *P. yoelii* CS. Mice immunized once or three times with *P. berghei* [PyCS] IrrSpz and then challenged with either *P. berghei* or *P. berghei* [PyCS] sporozoites were also protected (Figure 5B). However, since we detected antibody and T cell cross-reactive responses against long peptides derived from the *P. berghei* CS after immunization with *P. berghei* [PyCS] or *P. yoelii* IrrSpz (Figures 1 and 3), it was not possible to ascertain to what extent the anti-CS cross-reactive responses as opposed to the immune responses to non-CS antigens contributed to the sterile protection observed.

**Figure 6:**
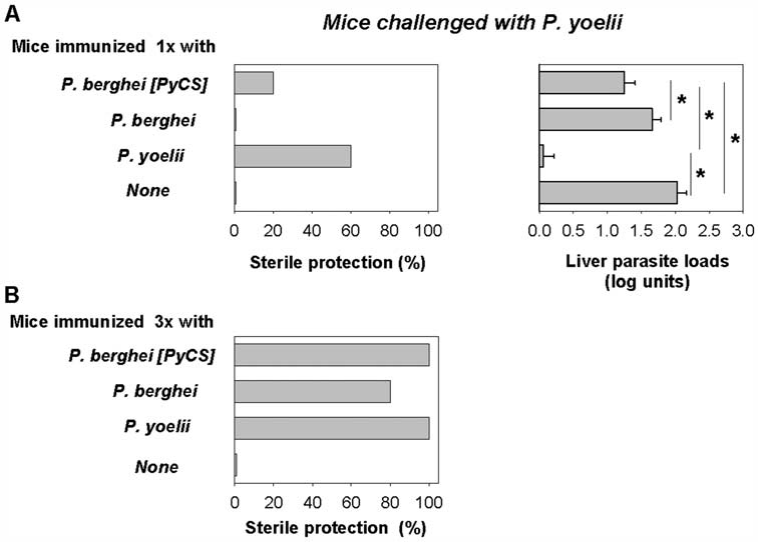
Sterile protection in mice immunized with IrrSpz from one or the other of the 3 different lines and challenged with *P. yoelii* sporozoites. Mice were immunized with 1 (**A**) or 3 (**B**) injections of the 3 different *Plasmodium* lines before challenge with *P. yoelii* sporozoites as described in the Materials and methods. Challenge was performed with 100 *P. yoelii* sporozoites 12–13 days after immunization with the single dose or one week after the last IrrSpz injection in the 3 immunizing dose regimen. Sterile protection (left panel) was determined after monitoring of all challenged groups (5 mice per group) for blood-stage infections by examination of Giemsa-stained blood smears obtained daily from day 3 to day 10 post-challenge. All naive control mice developed a patent blood-stage infection. Liver stage inhibition (**A**, right panel) was determined by measuring liver parasite development in mice immunized once with IrrSpz 42 hours after challenge with 60 000 *P. yoelii* sporozoites. Results were expressed as mean liver parasite load log units±SEM of 5 mice. Reduction of *P. yoelii* parasite load was more than 98% in *P. yoelii*-immunized animals, 83.4% in *P. berghei* [PyCS]-immunized animals and 57% in *P. berghei*-immunized animals when the arithmetic values were used for calculation. * *p*<0.05 (ANOVA followed by Tukey's test).

In order to address this point, mice were immunized once with IrrSpz from the 3 parasites lines and challenged with *P. yoelii* sporozoites. Sterile protection was obtained in 60% of the mice immunized with one injection of *P. yoelii* IrrSpz and challenged with homologous *P. yoelii* sporozoites but not in mice challenged with the heterologous *P. berghei* sporozoites (Figure 6A). On the other hand, 1 of the 5 mice immunized with *P. berghei* [PyCS] IrrSpz was completely protected against a *P. yoelii* sporozoite challenge (Figure 6A, left panel). This indicated that the presence of the *P. yoelii* CS in a *P. berghei* background could not account for sterile protection induced. Sterile protection is an all-or-none phenomenon that depends on maximal inhibition of parasite invasion and growth in the liver. Therefore, we quantified parasites in the livers of immunized and challenged mice in order to determine to what extent immunization with the 3 parasites lines inhibited the development of *P. yoelii* sporozoites. A single immunization with *P. yoelii* IrrSpz induced a significant 98.9% reduction of parasite liver load as compared to non-immunized mice (Figure 6A, right panel). Immunization with *P. berghei* IrrSpz reduced by 57% *P. yoelii* liver load as compared to non-immunized mice, a difference that did not reach statistical significance. When immunization was performed with *P. berghei* [PyCS] IrrSpz, hepatic development of challenge *P. yoelii* was significantly reduced (83.4%) as compared to non-immunized mice (Figure 6A, right panel). This indicated that PyCS in the context of *P. berghei* sporozoites did induce an immune response that inhibited *P. yoelii* liver stage development significantly. The contribution of the CS to liver stage inhibition was evident, but it was not possible to deduce a quantitative measure of this contribution to the overall inhibition, though simple subtraction indicated that this could be at least 30%.

We then performed experiments where the mice were immunized 3 times with IrrSpz before challenge with *P. yoelii* sporozoites. Complete or near-complete sterile immunity was observed for each combination (Figure 6B). The CS had no role in the cross-species sterile protection induced after 3 injections of *P. berghei* IrrSpz because immunization with *P. berghei* induced no cross-reactive immune response to the *P. yoelii* CS (Figures 1, 2 and 3). When we performed the reverse experiment, immunization with three doses of *P. yoelii* IrrSpz, which induced cross-reactive anti-CS immune responses, 66% of the immunized mice were fully protected from challenge with *P. berghei* sporozoites (Figure S7). However, immunization with a single dose of *P. yoelii* IrrSpz could not protect any of the BALB/c mice from a similar challenge. These observations indicated that in mice immunized with *P. yoelii* IrrSpz, a cross-reactive anti-CS immune response component contributes to cross-species sterile protection in addition to the non-CS cross-reactive one. However, the relative magnitude of these two components could not be deduced from these experiments with confidence.

## Discussion

More than forty years have passed since the demonstration that immunization with irradiated sporozoites induces sterile protection against a sporozoite challenge [2]. The majority of investigations aimed at elucidating these protective mechanisms, and at developing vaccines that reproduce them, has been based on the CS, a protein that was quickly discovered to make up the bulk of the proteins at the sporozoite surface [14] and to be the main target of antibody responses [4]. Two independent studies using distinct approaches have recently put the central role of CS in the acquisition of sterile immunity into question. The first based on mice made tolerant to the CS of *P. yoelii* provided indirect evidence for the role of other parasite antigens [8]. The second based on gene replacement in *P. berghei* provided conclusive evidence that sterile immunity can be induced independently of specific immune responses to CS [10]. Demonstration that immunization with the IrrSpz of one species can induce sterile protection against a sporozoite challenge by another was subsequently made [11], [12]. The possibility that non-CS antigens were implicated in cross-protection was raised, but a role for CS was favoured because of the relative sequence similarities between the CS of the two rodent malaria species used, *P. berghei* and *P. yoelii*. Furthermore, previous observations had shown that adoptive transfer of a T cell clone derived from *P. yoelii* IrrSpz-immunized mice and specific to the *P. yoelii* CS immunodominant CD8 epitope protected against a *P. berghei* sporozoite challenge [12]. In the studies presented here, we exploited gene replacement technology to investigate the role of the *P. yoelii* and *P. berghei* CS in the acquisition of sterile protection induced by IrrSpz immunization, and to ascertain to what extent immune responses to CS are implicated in the cross-species protection.

The data presented for immunization with *P. berghei* IrrSpz confirmed our previous conclusions that sterile protection was independent of immune responses specific to the *P. berghei* CS [10], and furthermore demonstrated that cross-species protection against a *P. yoelii* sporozoite challenge was equally independent of these same anti-CS immune responses. On the other hand, the conclusions from the reciprocal immunization, i.e. IrrSpz carrying the *P. yoelii* CS were less clear-cut. In this case a role for specific anti-CS immune responses in protection could not be dismissed, because we found evidence for their significant contribution to the inhibition of sporozoite invasion and development in hepatocytes both in vitro and in vivo. Although formal quantitative evaluation of this contribution was precluded, we estimated that it could plausibly account for up to 40% of the sterile protection observed after heterologous sporozoite challenge. This asymmetrical role for the CS in sterile protection was unexpected. This raises the possibility that a similar phenomenon might operate with the different parasites species that infect humans.

A possible explanation for our observations could be that the immune responses against CS and non-CS antigens are induced differentially in the two rodent malaria species. In our hands, the principal difference between the two model species lay in the number of IrrSpz injections that were required to induce sterile protection. Sterile protection in all animals was obtained after a single immunizing IrrSpz dose in the *P. berghei* model, whereas 2 to 3 doses were required to achieve the same level of protection in the *P. yoelii* model. We propose that for *P. berghei*, the immune responses against non-CS antigens, which are responsible for sterile protection, are induced rapidly following a single IrrSpz dose. By contrast, for *P. yoelii*, boosting with multiple IrrSpz doses would be required to achieve the levels of immune responses to non-CS proteins needed to confer sterile protection. Formal demonstration of this hypothesis must await the identification of these non-CS antigens.

The fact that the immune responses induced against CS in some models has little bearing on the acquisition of sterile immunity conferred by immunization with IrrSpz, should not be taken as basis to rule out inclusion of the CS alone or in combination with other antigens in vaccine formulation. First, others and we have shown that immune responses induced by various formulations against CS can significantly reduce liver stage development and even confer sterile immunity in immunized animals [15]–[18]. In humans, this has proven to be more difficult to achieve, but the induction of sterile immunity in half or more of the volunteers immunized by the RTS,S vaccine remains a very promising result [19]–[21]. Failure to achieve equivalent levels of sterile protection in adults and children living in African endemic areas [22]–[24] must be offset by the observations of reduced incidence of clinical malaria episodes in trials in Mozambique, Kenya and Tanzania [22], [25], [26].

It might be that the CS in Nature actually plays a role in immune evasion. The highly biased antibody responses to CS and its dominance on the sporozoite surface could lead to a monopolization of the antibody responses mounted by the host against sporozoites. In this way, the CS would deviate the host defences away from other antigens more apt at being targets of the sterile protective immunity, such as those induced by immunization with IrrSpz. In such as case, the identification of these non-CS antigens should be strongly encouraged, a point of view increasingly adopted by the community [27]. This antigen subset might also be implicated in the protective mechanisms that underlie the potent cross-species protection obtained through immunization with IrrSpz reported here and elsewhere [11]. The task of identifying these protective antigens will be facilitated by the availability of the entire genomic sequences of malaria parasites.

The prospect of inducing cross-species protection against malaria pre-erythrocytic stage, akin to that reported here, in humans is an exciting one. The perception that immunization with the irradiated *P. falciparum* or *P. vivax* sporozoites does not confer sterile protection against challenge with sporozoite of the heterologous species rests on observations made on a single volunteer immunized with sub-optimal doses of *P. falciparum* irradiated sporozoites, and who was not protected from a single subsequent *P. vivax* sporozoite challenge [28]. It would be judicious to undertake further trials of this nature in order to confirm or to refute the possibility that cross-species protective responses against the parasite's pre-erythrocytic stages can be acquired in humans. Indeed, the armamentarium to fight against malaria would be substantially enhanced, if it could be demonstrated that a single vaccine capable of protecting against the two most prevalent and pathogenic species of malaria could be developed. The recent exciting advances in the development of practical live sporozoite vaccination strategies [29]–[34] would make it possible to explore this strategy before elucidating the nature of the cross-species protective antigens.

## Materials and Methods

### Ethics Statement

All experiments and procedures involving mice were approved by the “Direction Departementale des Service Veterinaires de Paris, France (Authorisation No 75–129) and performed in compliance with regulations of the French Ministry of Agriculture for animal experimentation (1987).

### Construction of a Transgenic *P. berghei* Whose CS Gene Was Replaced by That of *P. yoelii*

This was done as depicted in Figure S2A. Briefly, plasmid pPyCS (cl9) was digested with *Apa* I + *Xba* I to release the targeting insert (∼8.7 kb) from the plasmid backbone. The insert was then purified, from a gel following electrophoresis, by phenol/chloroform extraction. Purified schizonts of a cloned line of *P. berghei* ANKA strain were transformed with 5–10 µg of targeting DNA using the Amaxa programme U33 and subsequently injected intraperitoneally (i.p.) into phenyl hydrazine-treated mice as described previously [13]. Pyrimethamine resistant parasites were selected in the TO mice as described previously [35], and cloned in mice by limiting dilution. *P. berghei* ANKA expressing the *P. yoelii* CS protein was referred to as *P. berghei* [PyCS]. Replacement was confirmed by DNA hybridisation (Figure S2B) and by immunostaining experiments (Figure S3). For the former, genomic DNA was isolated from parasites as previously described [13]. 5 µg of genomic DNA were digested with *Eco* RV, electrophoresed on 0.8% agarose gel and blotted onto nylon Hybond-N^+^ membrane (Amersham). The following DNA fragments were used as probes: a) 1.1 kb fragment amplified from the 3′ UTR sequence of the *PbCS* gene with the primers 3′UTR1CS (5′-ATA AAC ATT ACG CAT GAT TAT A) and 3′UTR2CS (5′-GAG TAC TCA CGA ATC CGA AAT AAG); and b) a 1.1 kb fragment of the *PyCS* gene with primers PyCS1( 5′-ATG AAG AAG TGT ACC ATT TTA GTT GTA GCG) and PyCS2 (5′-TTA ATT AAA GAA TAC TAA TAC). All hybridization experiments were carried out as described previously [13].

### Mice and *Plasmodium* Sporozoites

BALB/cJ and CD1 female mice were purchased from Harlan Laboratories (Gannat, France) and were housed in pathogen-free rodent barrier facility. *P. yoelii yoelii* 17XNL clone 1.1, a *P. berghei* ANKA cloned lined transfected with a GFP molecule derived from *P. berghei* ANKA clone 15cy1 and referred as *P. berghei* [36], two cloned lined of *P. berghei* [PyCS], which had been submitted to the same selecting procedure as that use to obtain the *P. berghei* GFP parasites were used to infect laboratory-bred *Anopheles stephensi* mosquitoes. The infectivity and development of *P. berghei* [PyCS] has been shown previously to be similar to those of the parent *P. berghei* both in the mosquito and in the mouse [13]. Sporozoites from the different lines were obtained by dissection of the salivary glands of the infected *A. stephensi* female mosquitoes 15 to 21 days after the infective blood meal.

### Immunization, Challenge and Protection Assessment

Mice were immunized intravenously with one single dose of 75 000 sporozoites or with one dose of 75 000 sporozoites followed by two booster doses of 25 000 sporozoites, in all cases irradiated at 12 000 rads, 15 and 22 days after the priming injection. Naïve control mice and mice immunized with irradiated sporozoites were challenged i.v. with 100 sporozoites of *P. yoelii* or 5 000 sporozoites of *P. berghei* 12–13 days after the single dose immunization or one week after the last IrrSpz injection in the 3 immunizing dose regimen. Because of differences in the infectivity of these *Plasmodium* species, the doses were chosen so as to induce infection in all control mice. Infection was determined by the presence of parasites in Giemsa-stained blood smears prepared daily from days 3 to 10 post-challenge and parasitaemia was determined by counting the number of infected red blood cells per 1000 erythrocytes. Quantification of parasite load in the liver of sporozoites-infected mice was made from a previous method [37] adapted to real time PCR. Mice were injected i.v. with 60 000 sporozoites. Forty-two to forty-four hours after, a liver biopsy was collected and DNA was extracted using the DNAeasy kit, including all optional steps (Qiagen, The Netherlands). At that time, liver parasite maturation is nearly complete but merozoites are yet to be released from the hepatocytes to initiate a blood stage infection as previously demonstrated using a sensitive nested PCR technique [38]. DNA quantity and quality were assessed by densitometry using a Nanodrop (ThermoFischer Scientific). The solution was adjusted to 10 ng/µl with water. Then, 50–100 ng of each sample were used as template for a real time quantitative PCR using the Lightcycler FastStart DNA Master SYBR Green I kit (Roche, Germany) in a Lightcycler (Roche, Germany) in duplicate. The primers used were NYU-Py3 (F) 5′- GGGGATTGGTTTTGACGTTTTTGCG-3′ and NYU-Py5(R) 5′- AAGCATTA AATAAAGCGAATACATCCTTAT-3′ [39], for *P. yoelii* and IC-PbF (5′-GAATTGGTTTTGACGTTTATGTGGGC-3′) and IC-PbR(5′ AAGCATTAAATAAAGCGAATACATCCTTAC-3′) for *P. berghei* which target the parasite's small subunit ribosomal RNA gene (ssrRNA). PCR conditions were as follows: SYBR green mix as indicated by manufacturer, final concentrations of 3 mM (for *P. yoelii* primers) or 3.5 mM (for *P. berghei* primers) MgCl_2_, 400 nM primers (F and R) in a total volume of 20 µl. The program used for amplification was: 95°C for 10 minutes, followed by 40 cycles of 95°C 10 seconds, 60°C 10 seconds and 72°C 10 seconds. The melting curve was generated by a linear increase of temperature from 67 to 90°C at 0.2°C/second. Standard curves were generated using a 10-fold dilution series (from 10^6^ to 1 parasites/µl) of DNA solution purified from blood stages of either *P. yoelii*, or *P. berghei* ANKA obtained from a sample in which the number of parasite nuclei/µl was determined accurately, by microscopy examination of Giemsa-stained blood smears and calculation of the number of RBC/µl of blood. Genomic DNA rather than a plasmid bearing a ssrRNA gene was used to generate the standard curve, because it reflects more accurately the multiple targets amplified since there are more than 5 different ssrRNA genes in the genome of *Plasmodium.* One liver parasite load unit corresponds to the log number of the parasite nuclei/µg of liver DNA. Sensitivity of the reaction allowed a linear detection down to 10 nuclei (slope of linear regression: −3.495) of parasites/100 ng liver DNA for both *P. berghei* and *P. yoelii*.

### Peptides

Peptides Py3 [(QGPGAP)_3_] and Pb2 [(DPPPPNPN)_2_], corresponding to the repeat regions of *the* CS protein of *P. yoelii* and *P. berghei*, respectively, were used in ELISA as previously described [18], [40]. The following peptides: PyB1 (SYVPSAEQI), PyB1L (SYVPSAEQILEFVKQIS), containing the dominant H2-K^d^ restricted CD8^+^ T cell epitopes in the *P. yoelii* CS [6], [41], PbB1 (SYIPSAEKI) and PbB1L (SYVPSAEKILEFVKQISSQ) containing the H2-K^d^ restricted CD8^+^ T cell epitopes in the *P. berghei* CS [5], [6], [12] were used in ELISPOT assays. Lyophilized material was resuspended in sterile distilled water at 10 mg/ml, aliquoted, and stored at −20°C until use. The following long peptides corresponding to NH_2_-terminal and COOH-terminal parts of the two different CS were kindly given by Giampietro Corradin (Institute of Biochemistry, University of Lausanne): *P. yoelii* CS long peptides (PyLN), PyNt (N-terminal region, amino acid segment 20–138: PGYGQNKSVQ AQRNNLYENN LHL SNGKINR NIVNRLLGDA NGKPEEKKDD PPKDGNKDDL PKEEKKDLPK EEKK DDPPKD PKKDDPPKNED) and PyCt (C-terminal region, amino acid segment 277–345: NEDSYVPSAE QIL EFVKQIS SQLTEEWSQC SVTCGSGVRV KRKNVNKQPE NLTLEDIDTE ICKMDKCS); *P. berghei* long peptides (PbLP), PbNt (amino acid segment 21–91: YGQNKSIQAQ RNLNELCYNE GNDNKLYHVL NSKNGKIYIR NTVN RLLADA PEGKKNEKKN KIERNNKLK) and PbCt (amino acid segment 242–310: NDDSYIP SAEKILEFVKQI RDSITEEWSQ CNVTCGS GIRVRKRKG SNKKAED LTLEDID TEICKMDKCS)[42]; In one experiment, PyLP were used to immunize mice in order to obtain specific antibodies to these regions for use in immunofluorescence assays.

### ELISPOT Assay

PVDF microplates (Millipore, Bedford, MA, USA) were coated overnight at 4°C with 15 µg/ml of an anti-mouse IFN-g rat mAb (clone AN18, Mabtech AB, Sophia Antipolis, France) diluted in PBS. After extensive washes and 2 hours-incubation at 37°C with RPMI medium containing 10% foetal calf serum, 3×10^5^ spleen cells were incubated overnight with the different peptides (final concentration 10 µg/ml) and with 30 U/ml of recombinant human IL-2. The plates were then washed, incubated with 2 µg/ml of biotinylated anti-mouse IFN-g rat monoclonal antibody (clone R4-6A2, Mabtech AB) diluted in PBS containing 0.5% bovine serum albumin for 2 h at 37°C, and then overnight at 4°C. Plates were subsequently incubated with extravidin-coupled alkaline phosphatase (Sigma-Aldrich) diluted in PBS. After adding the BCIP/NBT substrate (Sigma-Aldrich), IFN-g spot forming cells were counted under a stereomicroscope and expressed as the number of spots per million tested cells.

### ELISA

The presence and level of antibodies to Py3 and Pb2 peptides and to PyLP and PbLP were detected by ELISA as described previously [18], [40]. Briefly, 96-well flat-bottom plates (Maxisorp, Nunc, Roskilde, Denmark) were coated with 1 µg/ml of peptide in PBS, pH 7.8, by overnight incubation at 4°C. After extensive washes, and a 1 hour-incubation with 200 µl of PBS containing 0.05% Tween and 1% BSA, wells were incubated for 1 hour at 37°C with 100 µl of mouse sera diluted 1/100 in PBS-Tween-BSA. After two washes, wells were incubated for 45 min at room temperature, either with goat IgG anti-mouse IgM (Invitrogen SARL, Cergy Pontoise, France) or with a biotinylated goat anti-mouse IgG (Jackson ImunoResearch Europe Ltd, Newmarket, United Kingdom) diluted in PBS-Tween. The wells containing the goat IgG anti-IgM antibody were washed and further incubated with a biotinylated rabbit anti-goat IgG (Sigma-Aldrich, Saint-Quentin Fallavier, France) diluted in PBS-Tween for 45 min at room temperature, then washed and incubated with extravidin-coupled alkaline phosphatase (Sigma-Aldrich) diluted in PBS-Tween for 1 h at room temperature. Phosphatase activity was measured using 4-methylumbelliferyl phosphate (Sigma-Aldrich) as a substrate and the fluorescence at 355/460 nm was measured using a spectrophotometer (Victor 1420, Wallac Oy, Turku, Finland).

### Immunofluorescence Assay (IFA)

Sera from mice immunized with irradiated sporozoites were tested by immunofluorescence using wet or air-dried methanol-fixed sporozoites from the different *Plasmodium* lines, in order to detect surface or total antigen content as described previously [43].

### Sporozoite Invasion and Development Inhibition Assay

Human hepatoma cells, Hep-G2-CD81 (8×10^4^ cells/well) [44], which are fully susceptible to *P. yoelii* and *P. berghei* sporozoites, were cultured in eight-chamber plastic Lab-Teck slides (Nunc, Naperville, IL) in William's E medium (GIBCO, Edinburgh, Scotland) supplemented with 5% FCS (GIBCO), 1% penicillin-streptomycin solution (100X, stock solution, GIBCO) and incubated at 37°C in 3.5% C0_2_ for 24 hours. After removal of medium from the culture chambers, 10 000 sporozoites were added in 100 µl of fresh supplemented medium. Inhibition of sporozoite and liver stage development assay was performed as previously described [15]. Briefly, sera (1∶10 dilution) were added to hepatocyte cultures at the time of sporozoite inoculation and removed 3 hours later. Medium was replaced by fresh supplemented medium. Cultures were fixed with cold methanol after 45 hours. Sera from control naive mice were used as control. Schizont numbers were assessed in triplicate cultures by immunofluorescence assay using antibodies against PyHSP70.1 that recognizes *P. yoelii* liver stages as previously described [45]. Percent inhibition was calculated by comparing the numbers of parasites in the experimental cultures with the numbers in control wells.

## Supporting Information

Figure S1 Alignment of protein sequences from the CSP sequences used in this study. CSP was amplified by PCR using primers flanking the 5′ and 3′ ends of the CSP gene(underlined in figure). Sequences of P. yoelii CSP (GenBank accession number: bankit1261217, GQ86230) and of P. berghei GFP CSP (GenBank accession number: bankit1261246,GQ862302) were obtained and compared. The P.yoelii CSP from Pb (PyCSP) was identical to the CSP from P.yoelii 1.1 (confirmed by sequencing). Pre-, post and repeat regions are highlighted in green, and differences in non-repeat regions are highlighted in yellow.
(0.25 MB TIF)

Figure S2 The P. berghei CS (PbCS) locus and the integration of pPyCS. A Map of the pPyCS construct and schematic representation of the WT and targeted PbCS locus. To direct the 5′ recombination event, a 1.1 kb 5′ UTR sequence (thin grey box) of PbCS (wide black box) was inserted in front of the 1.1 kb PyCS coding region (wide white box). A 302 bp sequence corresponding to the PbCS 3′ UTR (thin white box) was placed downstream of PyCS. A further 848 bp of the PbCS 3′ UTR (thin white box) was inserted downstream of the DHFR-TS transcription unit (hatched box). The relative position of Eco RV (E) cleavage sites is indicated. Thick black lines (a, b) indicate the positions of the probes used in Southern blot experiments. B. Southern blot analyses of the parasites. Genomic DNA from WT and transgenic PyCS-5 parasites was digested with Eco RV and hybridized with the 2 different probes (a, b) to ascertain the correct integration of the constructs. Size markers are in kilobases (kb). The integrity of the inserted DNA fragment was also confirmed by PCR and sequence analysis (data not shown). These analyses demonstrated that the targeting construct (Figure S2A, panel a) had correctly integrated in the transgenic parasite thereby placing the PyCS coding sequence under the control of the P. berghei CS regulatory sequences and directing the downstream insertion of the selectable marker DHFR-TS (Figure S2B, panel b).
(0.67 MB TIF)

Figure S3 Antibodies to different regions of P. yoelii or P. berghei CS recognize homologous but not heterologous CS on sporozoites. Monoclonal antibodies specific to the repeat regions of the P. yoelii yoelii 17XNL (NYS1) (3) or the P. berghei ANKA (3.28) (4) CS and polyclonal antibodies (1/100 dilution) against the N-terminal or the C-terminal regions of the P. yoelii yoelii 17XNL CS were tested by IFA on dried methanol fixed sporozoites. Antibodies directed against the repeats or the flanking region of the P. yoelii CS recognized only P. yoelii and P. berghei [PyCS] but not P. berghei sporozoites. Antibodies to the repeat regions of P. berghei CS recognized only P. berghei parasites. References: (1)Charoenvit, Y. et al. 1987. Characterization of Plasmodium yoelii monoclonal antibodies directed against stage-specific sporozoite antigens. Infect Immun 55: 604–608. (2)Weber, J. L. et al.1987. Plasmodium berghei: cloning of the circumsporozoite protein gene. Exp Parasitol 63: 295–300.
(0.27 MB TIF)

Figure S4 IgM antibody responses to P. yoelii and P. berghei CS domains. Pooled serum samples from groups of mice immunized with the different parasite lines were analyzed by ELISA against different domains of the P. berghei (A), and P. yoelii (B) CS, using secondary antibodies specific to the IgM isotypes. Data are expressed as differential absorbance where values from pooled normal serum were subtracted from experimental values.
(0.10 MB TIF)

Figure S5 Antibody reactivity to dried methanol-fixed sporozoites induced by immunization with irradiated sporozoites. IgG response is exclusively directed against the CS. Individual serum samples from groups of mice immunized with the sporozoites from the different parasite lines were analyzed by IFAT against dried and methanol-fixed sporozoites to detect the total CS and other antigens content using secondary antibodies specific to IgG. Titres are expressed as the Mean±SD of the log of the highest dilution of serum that gave a positive staining.
(0.27 MB TIF)

Figure S6 Sterile protection in outbred CD1 mice immunized with P. berghei irradiated sporozoites and challenged with P. berghei or P. berghei [PyCS] sporozoites. CD1 mice were immunized with 3 injections of P. berghei and challenged with 5 000 sporozoites of P. berghei or P. berghei [PyCS]. All groups (5 mice per group) were monitored for blood-stage infections by examination of Giemsa-stained blood smears obtained daily from day 3 to day 10 post-challenge. All naive control mice developed a patent blood-stage infection.
(0.09 MB TIF)

Figure S7 Sterile protection in mice immunized with P. berghei irradiated sporozoites and challenged with P. yoelii. Mice were immunized either with a 1 injection or 3 injections of P. yoelii IrrSpz as described in the Materials and methods. Challenge was performed with 5 000 P. berghei sporozoites one week after the last IrrSpz injection. All groups were monitored for blood-stage infections by examination of Giemsa-stained blood smears obtained daily from day 2 to day 11 post-challenge. All naive control mice developed a patent blood-stage infection. The data represent pooled results from two experiments (with four to five mice per group in each experiment).
(0.09 MB TIF)
