# Supplementary figures and images for: A Role for Immune Responses against Non-CS Components in the Cross-Species Protection Induced by Immunization with Irradiated Malaria Sporozoites
Source: PLoS One. 2009 Nov 5;4(11):e7717. doi: 10.1371/journal.pone.0007717 (PMC2766644; doi:10.1371/journal.pone.0007717)

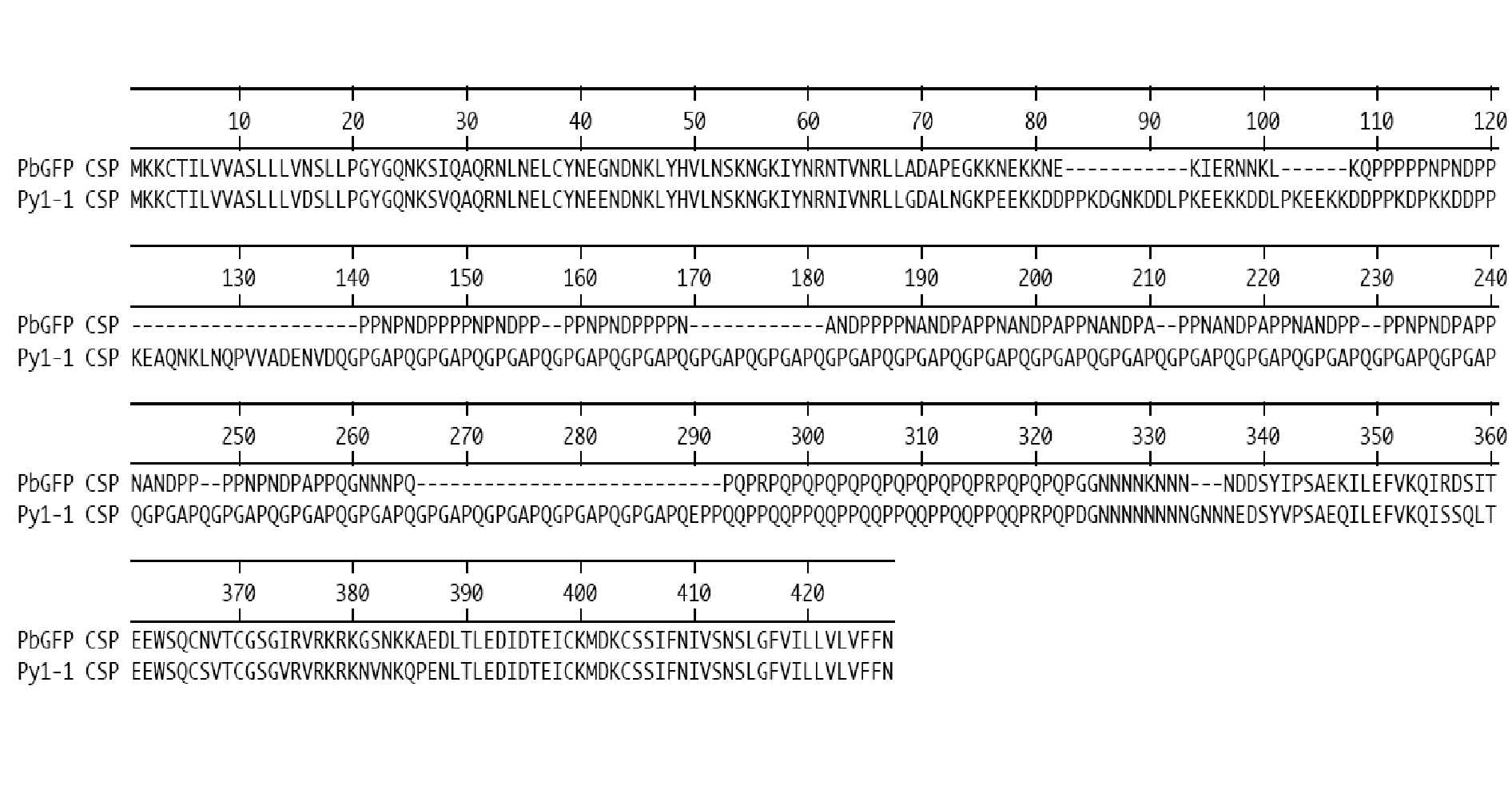

Supplement: Figure S1 — Alignment of protein sequences from the CSP sequences used in this study. CSP was amplified by PCR using primers flanking the 5′ and 3′ ends of the CSP gene(underlined in figure). Sequences of P. yoelii CSP (GenBank accession number: bankit1261217, GQ86230) and of P. berghei GFP CSP (GenBank accession number: bankit1261246,GQ862302) were obtained and compared. The P.yoelii CSP from Pb (PyCSP) was identical to the CSP from P.yoelii 1.1 (confirmed by sequencing). Pre-, post and repeat regions are highlighted in green, and differences in non-repeat regions are highlighted in yellow. (0.25 MB TIF) [file pone.0007717.s001.tif]

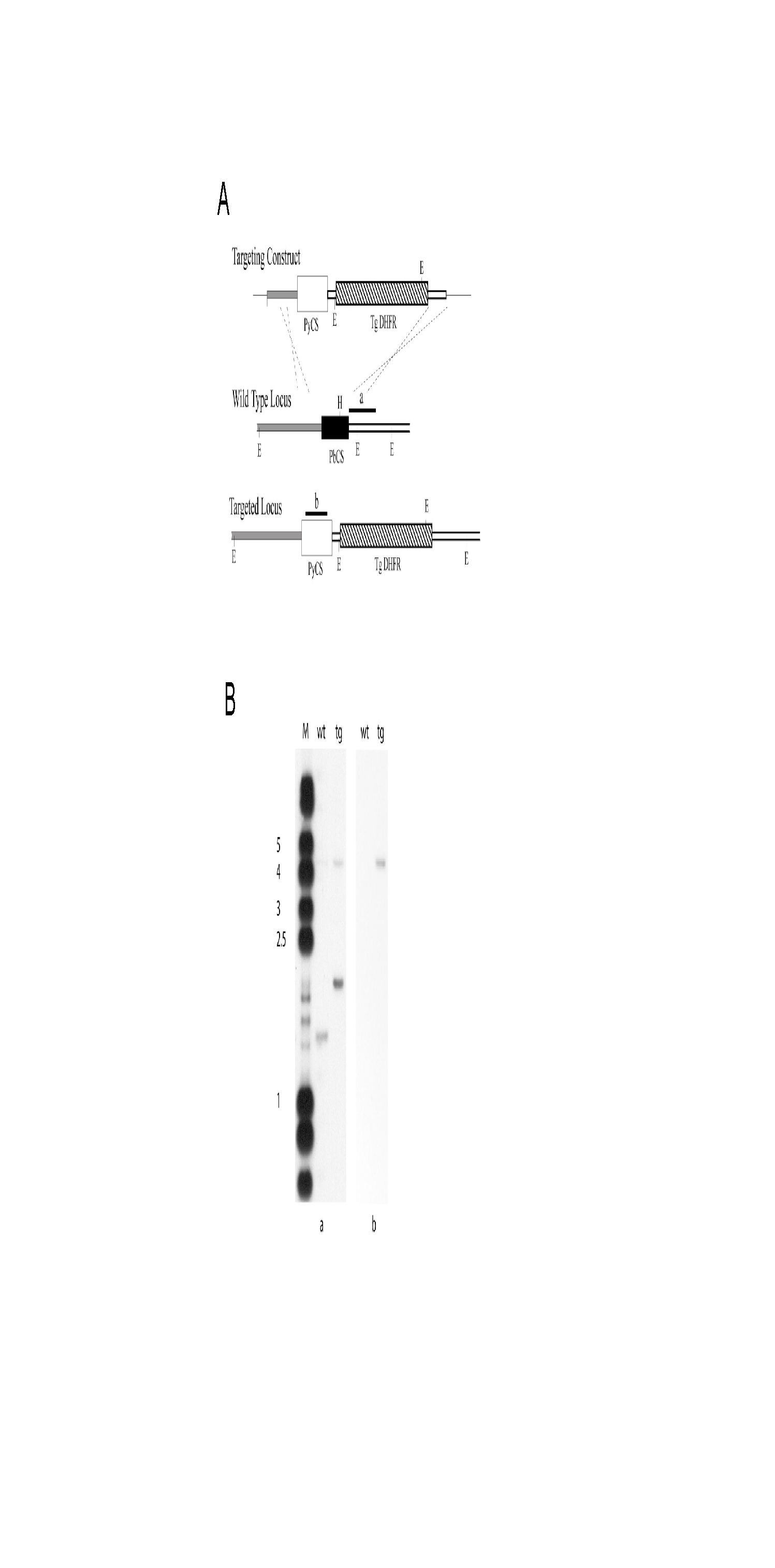

Supplement: Figure S2 — The P. berghei CS (PbCS) locus and the integration of pPyCS. A Map of the pPyCS construct and schematic representation of the WT and targeted PbCS locus. To direct the 5′ recombination event, a 1.1 kb 5′ UTR sequence (thin grey box) of PbCS (wide black box) was inserted in front of the 1.1 kb PyCS coding region (wide white box). A 302 bp sequence corresponding to the PbCS 3′ UTR (thin white box) was placed downstream of PyCS. A further 848 bp of the PbCS 3′ UTR (thin white box) was inserted downstream of the DHFR-TS transcription unit (hatched box). The relative position of Eco RV (E) cleavage sites is indicated. Thick black lines (a, b) indicate the positions of the probes used in Southern blot experiments. B. Southern blot analyses of the parasites. Genomic DNA from WT and transgenic PyCS-5 parasites was digested with Eco RV and hybridized with the 2 different probes (a, b) to ascertain the correct integration of the constructs. Size markers are in kilobases (kb). The integrity of the inserted DNA fragment was also confirmed by PCR and sequence analysis (data not shown). These analyses demonstrated that the targeting construct (Figure S2A, panel a) had correctly integrated in the transgenic parasite thereby placing the PyCS coding sequence under the control of the P. berghei CS regulatory sequences and directing the downstream insertion of the selectable marker DHFR-TS (Figure S2B, panel b). (0.67 MB TIF) [file pone.0007717.s002.tif]

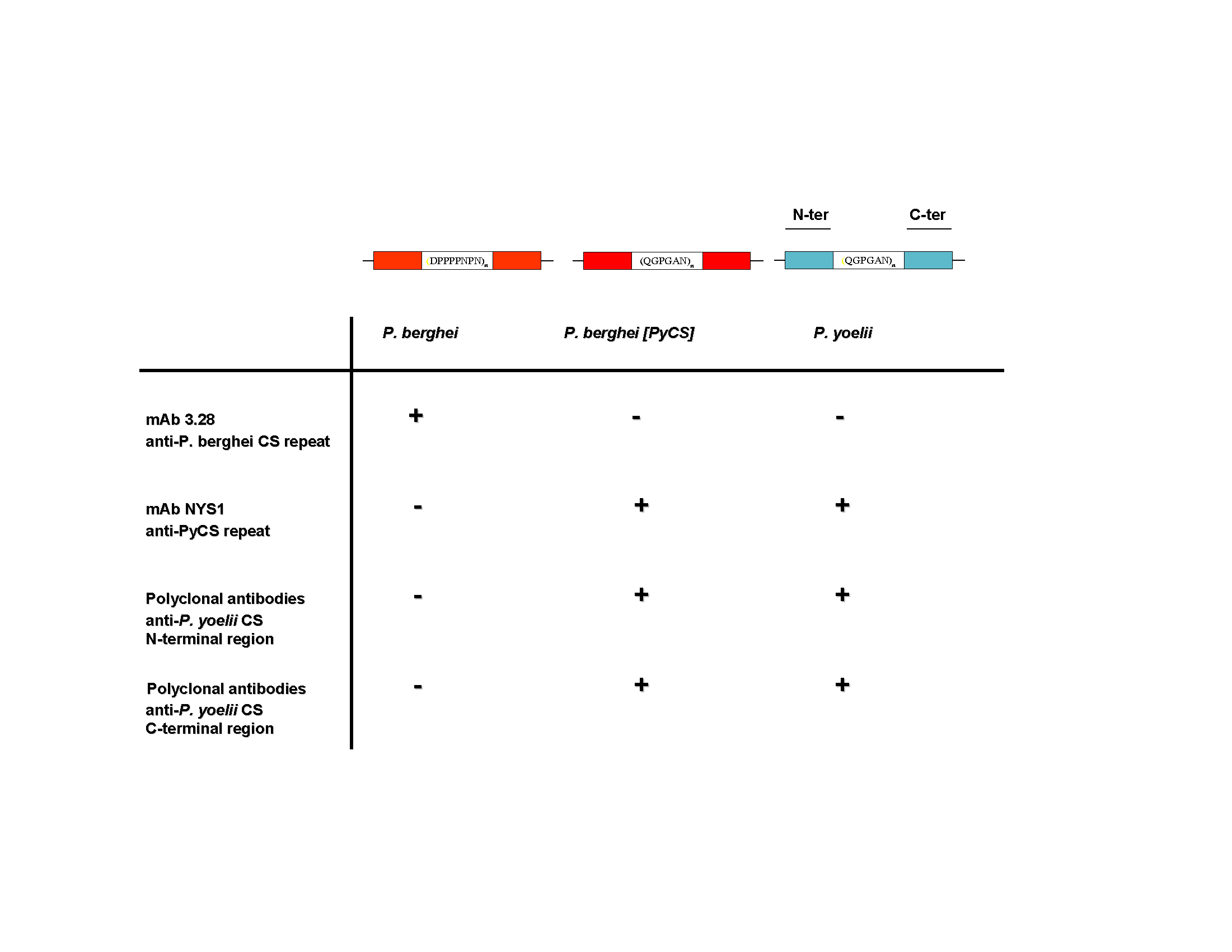

Supplement: Figure S3 — Antibodies to different regions of P. yoelii or P. berghei CS recognize homologous but not heterologous CS on sporozoites. Monoclonal antibodies specific to the repeat regions of the P. yoelii yoelii 17XNL (NYS1) (3) or the P. berghei ANKA (3.28) (4) CS and polyclonal antibodies (1/100 dilution) against the N-terminal or the C-terminal regions of the P. yoelii yoelii 17XNL CS were tested by IFA on dried methanol fixed sporozoites. Antibodies directed against the repeats or the flanking region of the P. yoelii CS recognized only P. yoelii and P. berghei [PyCS] but not P. berghei sporozoites. Antibodies to the repeat regions of P. berghei CS recognized only P. berghei parasites. References: (1)Charoenvit, Y. et al. 1987. Characterization of Plasmodium yoelii monoclonal antibodies directed against stage-specific sporozoite antigens. Infect Immun 55: 604–608. (2)Weber, J. L. et al.1987. Plasmodium berghei: cloning of the circumsporozoite protein gene. Exp Parasitol 63: 295–300. (0.27 MB TIF) [file pone.0007717.s003.tif]

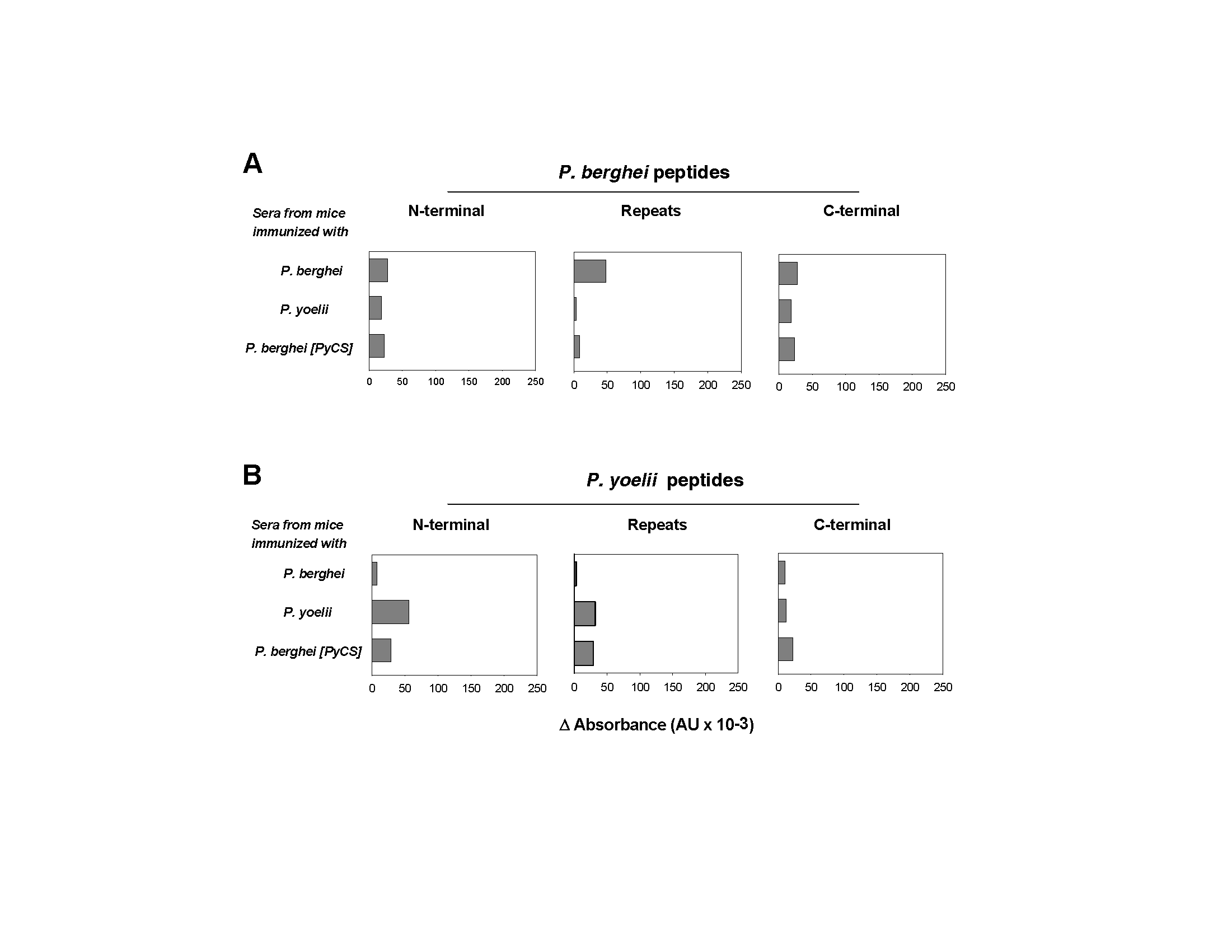

Supplement: Figure S4 — IgM antibody responses to P. yoelii and P. berghei CS domains. Pooled serum samples from groups of mice immunized with the different parasite lines were analyzed by ELISA against different domains of the P. berghei (A), and P. yoelii (B) CS, using secondary antibodies specific to the IgM isotypes. Data are expressed as differential absorbance where values from pooled normal serum were subtracted from experimental values. (0.10 MB TIF) [file pone.0007717.s004.tif]

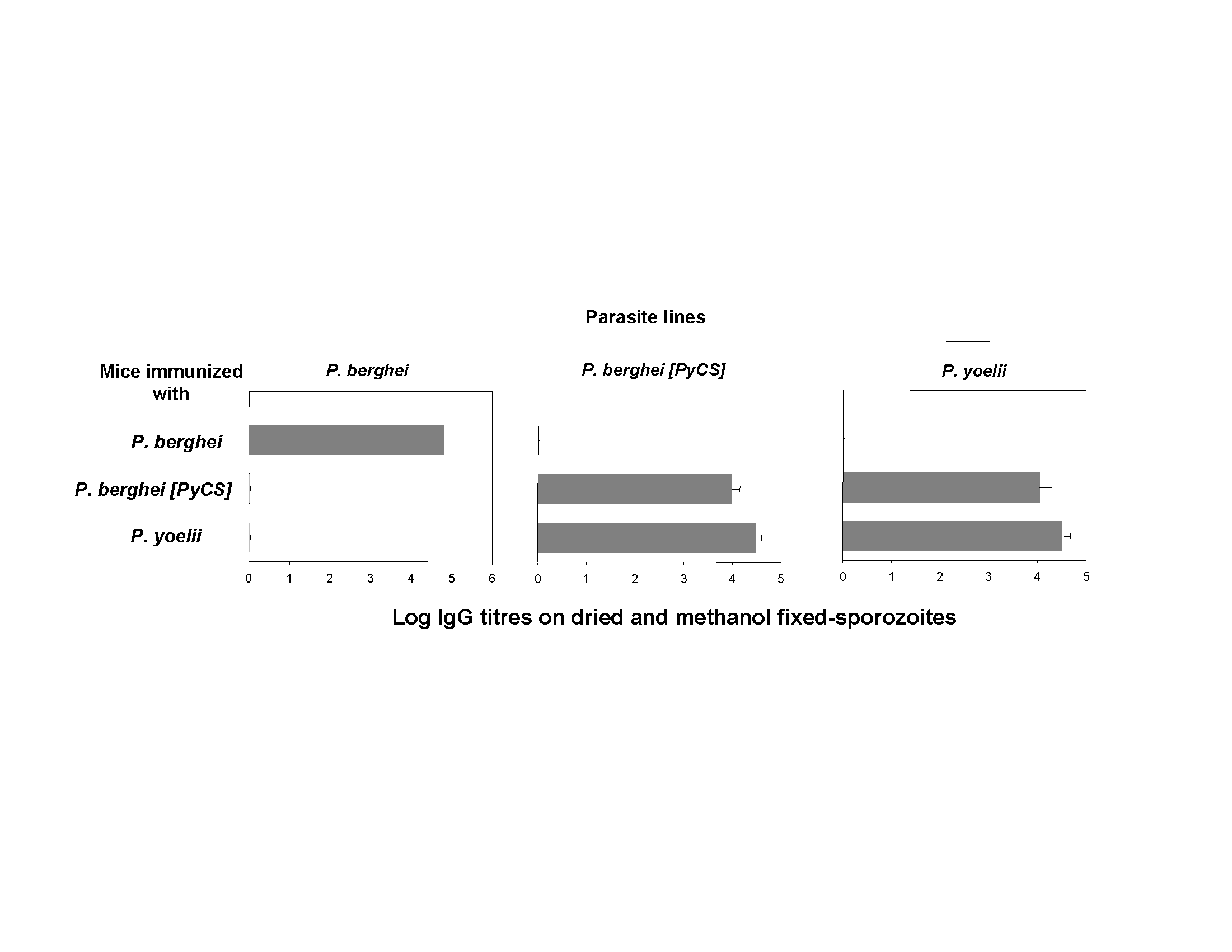

Supplement: Figure S5 — Antibody reactivity to dried methanol-fixed sporozoites induced by immunization with irradiated sporozoites. IgG response is exclusively directed against the CS. Individual serum samples from groups of mice immunized with the sporozoites from the different parasite lines were analyzed by IFAT against dried and methanol-fixed sporozoites to detect the total CS and other antigens content using secondary antibodies specific to IgG. Titres are expressed as the Mean±SD of the log of the highest dilution of serum that gave a positive staining. (0.27 MB TIF) [file pone.0007717.s005.tif]

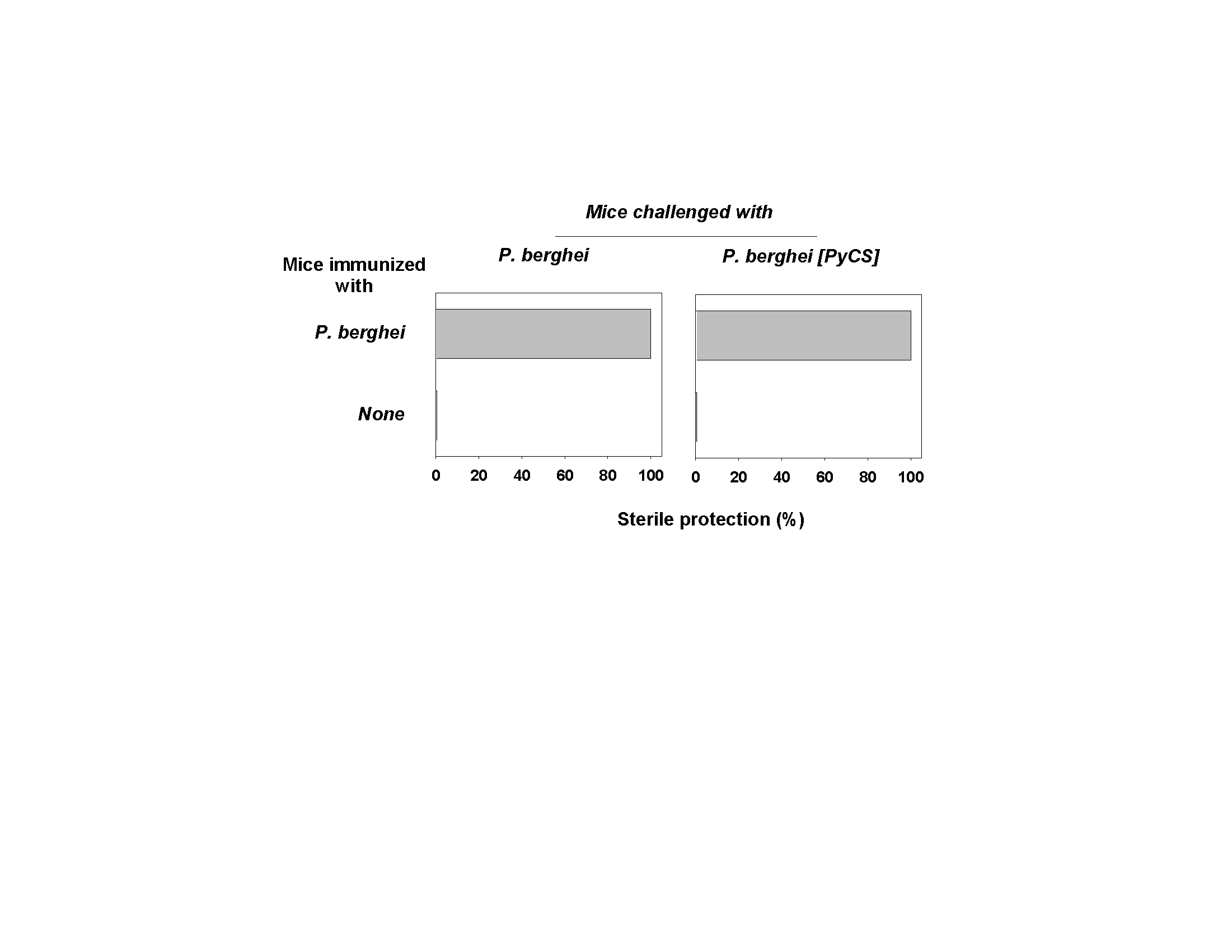

Supplement: Figure S6 — Sterile protection in outbred CD1 mice immunized with P. berghei irradiated sporozoites and challenged with P. berghei or P. berghei [PyCS] sporozoites. CD1 mice were immunized with 3 injections of P. berghei and challenged with 5 000 sporozoites of P. berghei or P. berghei [PyCS]. All groups (5 mice per group) were monitored for blood-stage infections by examination of Giemsa-stained blood smears obtained daily from day 3 to day 10 post-challenge. All naive control mice developed a patent blood-stage infection. (0.09 MB TIF) [file pone.0007717.s006.tif]

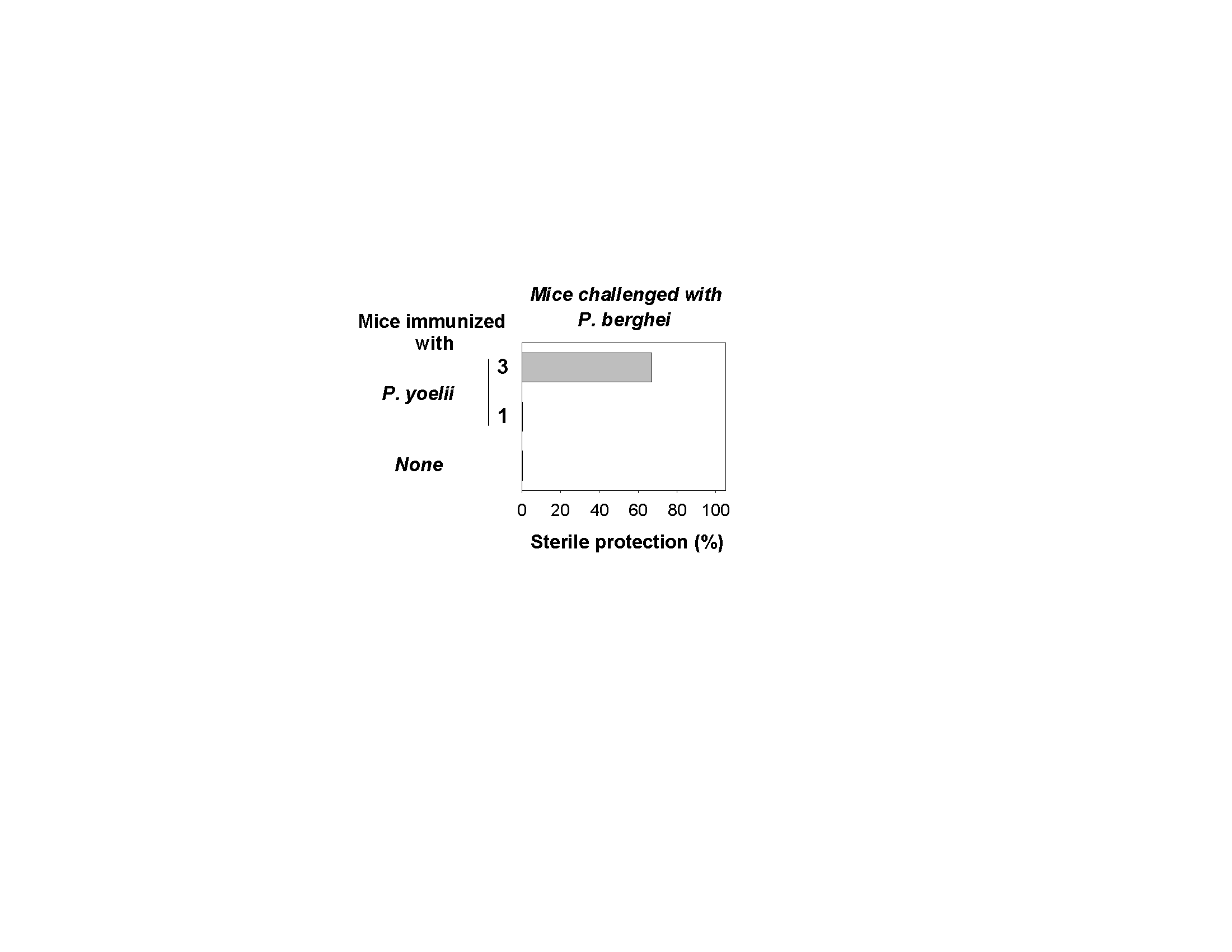

Supplement: Figure S7 — Sterile protection in mice immunized with P. berghei irradiated sporozoites and challenged with P. yoelii. Mice were immunized either with a 1 injection or 3 injections of P. yoelii IrrSpz as described in the Materials and methods. Challenge was performed with 5 000 P. berghei sporozoites one week after the last IrrSpz injection. All groups were monitored for blood-stage infections by examination of Giemsa-stained blood smears obtained daily from day 2 to day 11 post-challenge. All naive control mice developed a patent blood-stage infection. The data represent pooled results from two experiments (with four to five mice per group in each experiment). (0.09 MB TIF) [file pone.0007717.s007.tif]
